# Saline versus balanced crystalloids for hydration post-kidney biopsy

**DOI:** 10.1007/s00467-024-06594-0

**Published:** 2024-11-25

**Authors:** Yu Tanaka, Tomoko Horinouchi, Yuta Inoki, Yuta Ichikawa, Chika Ueda, Hideaki Kitakado, Atsushi Kondo, Nana Sakakibara, Shingo Ishimori, Tomohiko Yamamura, China Nagano, Kandai Nozu

**Affiliations:** Department of Pediatrics, Kobe University Graduate School of Medicine, 7-5-1 Kusunoki-Cho, Chuo-ku, Kobe, Hyogo 650-0017 Japan

**Keywords:** Maintenance fluid therapy, 0.9% sodium chloride, Balanced crystalloids, Hyponatremia, Acidosis, Hyperchloremia

## Abstract

**Background:**

Isotonic fluids are becoming the standard for hydration and maintenance fluid therapy, but there is no consensus on the optional choice among the different types of isotonic solution.

**Methods:**

This study is a single-center, non-randomized controlled trial at Kobe University Hospital, Japan, between April 2021 and March 2023. The study included pediatric patients aged 1–19 years who underwent kidney biopsies. From April 2021 to March 2022, 0.9% sodium chloride (saline) was administered, and from April 2022 to March 2023, balanced crystalloids were used. The primary outcome was the occurrence of hyponatremia (< 137 mEq/L) after a kidney biopsy. Secondary outcomes included other electrolyte balances, blood gas parameters, creatinine-based estimated glomerular filtration rate (Cr-eGFR), and arginine vasopressin concentrations (UMIN Clinical Trial Registry: UMIN 000044330).

**Results:**

Of 61 patients enrolled, 2 were excluded, leaving 34 in the saline group and 25 in the balanced crystalloid group. No hyponatremia occurred, and serum sodium concentrations were similar between both groups (138.7 vs. 138.9 mEq/L, *P* = 0.08). The saline group showed a greater increase in serum chloride (+ 1.7 vs. + 0.2, *P* < 0.01) and a greater decrease in HCO_3_^−^ concentrations (− 0.6 vs. + 0.9, *P* < 0.01). There were minimal changes in pH (− 0.01 vs. − 0.01,* P* = 0.99) and Cr-eGFR (− 1.5 vs. + 1.1 mL/min/1.73 m^2^, *P* = 0.96) in both groups.

**Conclusions:**

During pediatric kidney biopsy, both saline and balanced crystalloids were effective in preventing hyponatremia. Although saline infusion results in higher serum chloride concentrations and lower blood HCO_3_^−^ concentrations than balanced crystalloids infusion, the clinical significance was minimal.

**Graphical abstract:**

**Figure:**
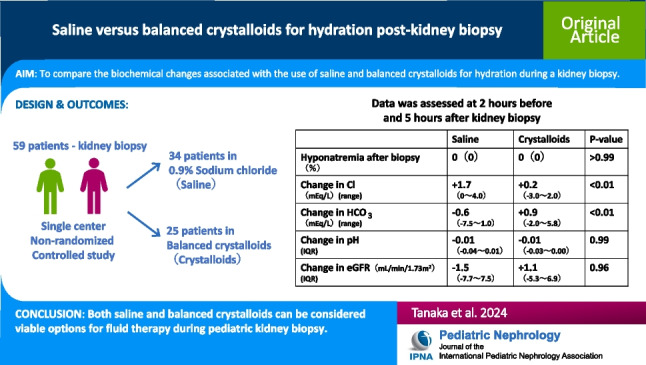
A higher resolution version of the Graphical abstract is available as Supplementary information

**Supplementary Information:**

The online version contains supplementary material available at 10.1007/s00467-024-06594-0.

## Introduction

In pediatric medicine, intravenous fluid therapy is one of the most common treatments. However, uncertainty remains regarding the optimal treatment strategy for this therapy. Recently, fluid therapy for children typically involves the use of isotonic and hypotonic solutions. Isotonic solutions, such as 0.9% sodium chloride (saline) and balanced crystalloids, match the tonicity of extracellular fluids, while hypotonic solutions have lower tonicity than extracellular fluids. Traditional maintenance fluid therapy, initially proposed by Holliday and Segal, has relied on hypotonic solutions [1]. However, there have been reports of hyponatremia-induced encephalopathy or even death following the administration of hypotonic solutions in pediatric care [2, 3]. Serum sodium concentrations are regulated by the renin–angiotensin–aldosterone system, which plays a role in sodium reabsorption, as well as by arginine vasopressin (AVP), which controls free water excretion. Causes of stimulation of AVP secretion can be classified into osmotic stimuli, such as volume depletion, hypotension, congestive heart failure, cirrhosis, nephrotic syndrome, and sepsis, as well as non-osmotic stimuli, such as pain, stress, nausea, vomiting, hypoxemia, hypercapnia, medications, inflammation, pulmonary disease, and central nervous system disease [1]. In the postoperative period, AVP secretion triggered by non-osmotic stimuli, such as pain, nausea, vomiting, stress, and medications, is the most common. In this situation, administering a hypotonic solution for maintenance fluid therapy can exacerbate hyponatremia because AVP impairs free water excretion [3–5]. Furthermore, isotonic fluid as maintenance fluid therapy has been reported to be effective in preventing hyponatremia.

We previously conducted a retrospective comparative study to ascertain the safety of hydration with hypotonic fluid and saline in pediatric patients after a kidney biopsy [6]. We found that administering a hypotonic fluid had a risk of hyponatremia. Regarding maintenance fluid therapy in children, in recent years, isotonic fluid has emerged as the standard solution, leading to a decreased likelihood of iatrogenic hyponatremia due to hypotonic solution [7–9]. However, the differences in safety among various isotonic solutions as hydration therapy remain unclear. Saline, which is the most used major maintenance fluid, is also associated with issues, such as hyperchloremia, acidosis, and kidney impairment [10]. Additionally, many previous studies on the safety of fluid therapy focused on acute settings, such as emergency care, the intensive care unit (ICU), and postoperative care [11–13]. However, in pediatric inpatients, frequent moderately invasive procedures involving mild sedation, such as lumbar puncture, bone marrow aspiration, liver biopsy, and kidney biopsy, are performed. During these procedures, many patients are not in an acute state but rather in a stable hemodynamic condition. However, there have been few studies on the safety of fluid therapy for these patients. We consider that determining the appropriate fluid infusion during such procedures is important. This study follows our previous investigation of the safety of hypotonic solution and saline during a kidney biopsy [6]. This study aims to compare the biochemical changes associated with the use of saline and balanced crystalloids as hydration during kidney biopsy in pediatric patients.

## Methods

### Study design

We conducted a single-center, non-randomized controlled trial to compare the use of saline with balanced crystalloids for intravenous fluid administration during kidney biopsies at the Pediatric Department of Kobe University Hospital, Japan, from April 2021 to March 2023. The trial received approval from the institutional review board at Kobe University (IRB number: B200377), and an informed consent for kidney biopsy was obtained from all participants. This trial was registered at the UMIN Clinical Trial Registry (UMIN 000044330), and we used the CONSORT checklist when writing our report.

### Patients and interventions

This study involved 61 pediatric patients aged 1–19 years who underwent percutaneous needle kidney biopsies at the pediatric ward. Patients who underwent a kidney biopsy between April 2021 and March 2022 received saline (saline group). Those who underwent this procedure between April 2022 and March 2023 received 5% glucose-containing acetic acid Ringer’s solution (with sodium 130 mEq/L, potassium 4 mEq/L, chloride 109 mEq/L, and acetate 28 mEq/L) as balanced crystalloids (balanced crystalloid group). Fluid administration started at a rate of 10 mL/hour 2 h before the biopsy (T0). After the biopsy, the rate was adjusted to 10 mL/kg/hour (with a maximum of 200 mL/hour) for the initial 2 h. Following this time, the rate was reduced to 5 mL/kg/hour (with a maximum of 100 mL/hour) for a duration of 3 h. Five hours after the biopsy (T7), blood tests were conducted, and the infusion rate was set at 3 mL/kg/hour (with a maximum of 50 mL/hour) (Supplementary Fig. 1). This hydration protocol used at our institution is original and specifically designed for post-kidney biopsy management. To prevent volume loss from bleeding and the formation of blood clots in the bladder, we administer an adequate amount of fluids following the biopsy. Additionally, increasing urine output helps facilitate the early detection of gross hematuria. Oral fluid intake was restricted starting 2 h before the kidney biopsy, and post-biopsy intake was permitted once the patient’s consciousness was fully confirmed. The kidney biopsy was performed at the pediatric ward using a 16-G biopsy needle. The number of kidney biopsies was determined by assessing the condition of the specimens collected. Sedation during this procedure comprised midazolam and pentazocine, with lidocaine administered as a local anesthetic. Narcotics and non-steroidal anti-inflammatory drugs were not administered. Blood samples were collected at two time points: at the start of hydration (T0) and 7 h after the initiation of hydration (T7) which was 5 h post-biopsy (Supplementary Fig. 1). The treatment details were open to the study investigators, treating physicians, nurses, and patients. Infusions were administered using electronic pumps, and the dosage was accurately adjusted according to the protocol. Blood samples were collected via venipuncture for laboratory testing. The results of lab data were extracted from medical records, and the presence or absence of postoperative nausea and vomiting (PONV) and pain after kidney biopsy was assessed based on patient interviews conducted by nurses.

### Outcomes

The primary outcome was the presence of hyponatremia at T7 and changes in sodium levels from T0 to T7. Secondary outcomes included plasma AVP concentrations, creatinine-based estimated glomerular filtration rate (Cr-eGFR), electrolyte concentrations, and blood gas parameters assessed at T7, as well as changes in these levels from T0 to T7. Additionally, the presence or absence of PONV and pain was assessed after kidney biopsy. To perform the calculation of the Cr-eGFR, we used an estimation formula specific to Japanese adults in patients aged 18 years and older [14], while a pediatric eGFR estimation formula specific to Japanese pediatric patients was used in those aged younger than 18 years [15]. Hyponatremia, hypokalemia, hyperchloremia, and hypersecretion of AVP were defined as serum sodium concentrations < 137 mEq/L, potassium concentrations < 3.5 mEq/L, chloride concentrations > 110 mEq/L, and AVP concentrations > 2.8 pg/mL according to the institutional testing criteria. In addition, metabolic acidosis was defined as a pH < 7.25 and an HCO_3_ < 22.0 mmol/L.

### Statistical analysis

Data of electrolytes and pH were presented as mean and range, while other parameters were reported as median and interquartile range (IQR). The Mann–Whitney rank-sum test or unpaired *t*-test was used as appropriate to analyze continuous variables. Nominal variables were analyzed using Fisher’s exact test. A significance level of *P* < 0.05 was considered statistically significant. All statistical analyses were performed using Easy R (EZR) [16], which is a statistical analysis software. EZR is a modified version of the R commander tailored to incorporate statistical functions commonly used in biostatistics.

## Results

### Baseline characteristics

**Fig. 1:**
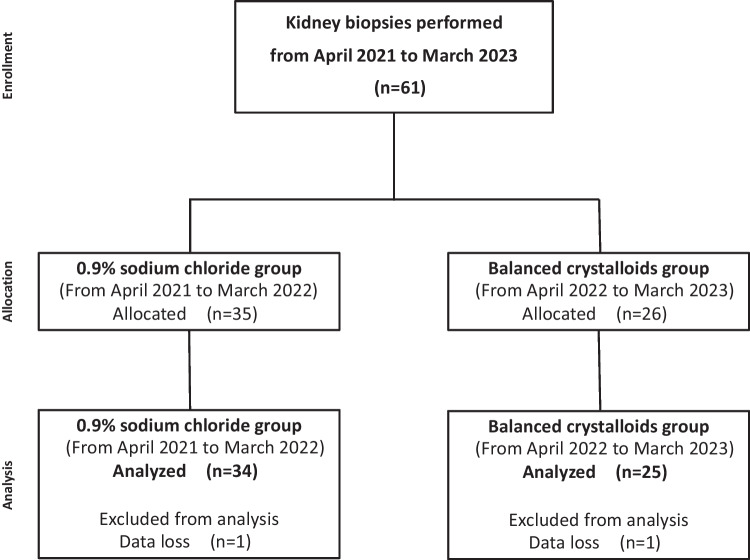
Study cohort selection. In this study, 61 cases that had kidney biopsies from 2021 to 2023 were registered. Due to missing data, one case from each group was excluded. Consequently, the final analysis included 34 cases in the 0.9% sodium chloride group and 25 cases in the balanced crystalloid group

Owing to missing data, 2 of the 61 patients were excluded. In the saline and balanced crystalloid groups, one patient in each group missed a blood gas test because of sample clotting. As a result, the final number of patients in the saline group was 34 and that in the balanced crystalloid group was 25 (Fig. 1). The indications for a kidney biopsy are shown in Table 1. The baseline clinical characteristics are shown in Table 2. The median age was 11.5 (7.3–15.0) years in the saline group and 12.0 (9.0–15.0) years in the balanced crystalloid group. There was no significant difference in the patients’ background, except for serum potassium concentrations, between the saline and balanced crystalloid groups. The mean serum potassium concentration in the saline group was higher than that in the balanced crystalloid group at T0 (4.3 vs. 4.1 mEq/L, *P* = 0.02).

**Table 1:** Indication for a kidney biopsy

	0.9% sodium chloride *n* = 34 (%)	Balanced crystalloids *n* = 25 (%)
Nephrotic syndrome	16 (47)	5 (20)
IgA nephropathy	6 (17)	7 (28)
Kidney transplantation	2 (6)	3 (12)
Asymptomatic proteinuria	3 (9)	0 (0)
TINU syndrome	1 (3)	4 (16)
Alport syndrome	2 (6)	0 (0)
SLE	1 (3)	1 (4)
Others AAV, CNI toxicity, HSPN, HUS, interstitial nephritis, mitochondria disease, nephronophthisis, Sjögren syndrome, CKD	3 (9)	5 (20)

*TINU*, tubulointerstitial nephritis and uveitis; *SLE*, systemic lupus erythematosus; *AAV*, antineutrophil cytoplasmic antibody–associated vasculitis; *CNI*, calcineurin inhibitors; *HSPN*, Henoch-Schönlein purpura nephritis; *HUS*, hemolytic-uremic syndrome; *CKD*, chronic kidney disease

**Table 2:** Baseline characteristics of patients

	0.9% sodium chloride (*n* = 34)	Balanced crystalloids (*n* = 25)	*P* value
Baseline			
Age (year)	11.5 (7.3− 15.0)	12 (9.0 − 15.0)	0.56
Female sex (*n*[%])	17 (50)	13 (52)	> 0.99
Weight (kg)	40.4 (24.2 − 51.0)	36.5 (28.0 − 50.4)	0.81
Height (cm)	141.0 (124.6 − 154.3)	148.9 (132.9 − 157.2)	0.58
Initial blood data			
Albumin (g/dL)	4.0 (3.7 − 4.3)	4.2 (3.9 − 4.4)	0.34
BUN/Cr	24.7 (17.5 − 29.5)	23.6 (13.7 − 30.3)	0.55
T0 Na (mEq/L)	138.7 (135.0 − 137.0)	138.7 (136.0 − 142.0)	0.90
T0 K (mEq/L)	4.3 (3.7 − 5.4)	4.1 (3.5 − 4.7)	0.02
T0 Cl (mEq/L)	106.2 (100.0 − 110.0)	106.0 (102.0 − 109.0)	0.65
T0 Na-Cl (mEq/L)	32.5 (26.0 − 37.0)	32.8 (30.0 − 35.0)	0.54
T0 pH	7.37 (7.314 − 7.471)	7.38 (7.316 − 7.500)	0.43
T0 HCO_3 _(mEq/L)	23.4 (16.9 − 26.7)	23.5 (19.3 − 28.0)	0.85
T0 eGFR (mL/min/1.73 m^2^)	104.3 (95.0 − 118.1)	97.6 (84.3 − 116.1)	0.52
T0 AVP (pg/mL)	0.8 (0.5 − 1.4)	1.2 (0.7 − 1.7)	0.42

*Cr*, creatinine; *eGFR*, estimated glomerular filtration rate; *AVP*, arginine vasopressin; *T0*, time zero

### Primary outcome

The clinical parameters following fluid therapy after a kidney biopsy are shown in Table 3. No cases of hyponatremia were observed, and there was no significant difference in serum sodium concentrations between the saline and balanced crystalloid groups at T7 (138.7 vs. 138.9 mEq/L, *P* = 0.08). Additionally, the mean change in serum sodium concentrations was not significantly different from T0 to T7 (+ 0.7 vs. + 0.1 mEq/L, *P* = 0.06).

**Table 3:** Result of blood test after kidney biopsies

	0.9% sodium chloride (*n* = 34)	Balanced crystalloids (*n* = 25)	*P* value
T7 Na (mEq/L)	138.7 (137 − 141)	138.9 (137 − 141)	0.08
T7 hyponatremia (%)	0 (0)	0 (0)	> 0.99
T7 K (mEq/L)	4.1 (3.5 − 5.0)	4.0 (3.5 − 5.3)	0.24
T7 hypokalemia (%)	0 (0)	0 (0)	> 0.99
T7 Cl (mEq/L)	107.9 (102.0 − 112.0)	106.2 (102.0 − 108.0)	< 0.01
T7 hyperchloremia (%)	27 (9/34)	4 (1/25)	0.03
T7 Na-Cl (mEq/L)	31.5 (27 − 36)	32.7 (29 − 36)	0.03
T7 pH	7.36 (7.27 − 7.49)	7.36 (7.33 − 7.46)	0.85
T7 HCO_3_ (mEq/L)	22.9 (16.7 − 27.3)	24.5 (19.3 − 29.6)	0.02
T7 eGFR (mL/min/1.73 m^2^)	99.3 (82.9 − 119.7)	90.4 (86.4 − 115.9)	0.46
T7 AVP (pg/mL)	1.0 (0.4 − 1.8)	1.1 (0.7 − 1.9)	0.31
T7 AVP > 2.8 pg/mL (%)	21 (7/34)	24 (6/25)	0.77
Change in Na (mEq/L)	0.7 (− 2.0 ~ 3.0)	0.1 (− 3.0 ~ 2.0)	0.06
Change in Cl (mEq/L)	1.7 (0 ~ 4.0)	0.2 (− 3.0 ~ 2.0)	< 0.01
Change in HCO_3_ (mEq/L)	− 0.6 (− 7.5 ~ 1.0)	0.9 (− 2.0 ~ 5.8)	< 0.01
Change in pH	− 0.01 (− 0.05 ~ 0.08)	− 0.01 (− 0.03 ~ 0.00)	0.99
Change in eGFR (mL/min/1.73 m^2^)	− 1.5 (− 7.7 ~ 7.6)	1.1 (− 5.3 ~ 6.9)	0.96

*eGFR*, estimated glomerular filtration rate; *AVP*, arginine vasopressin; *T7*, time + 7 h

### Secondary outcomes

Serum chloride concentrations were significantly higher in the saline group than in the balanced crystalloid group at T7 (107.9 vs. 106.2 mEq/L, *P* < 0.01). HCO_3_^−^ concentrations were significantly lower in the saline group than in the balanced crystalloid group at T7 (22.9 vs. 24.5 mEq/L, *P* = 0.02). When we focused on the changes from T0 to T7, we found that serum chloride concentrations showed a greater increase (+ 1.7 vs. + 0.2 mEq/L, *P* < 0.01) and HCO_3_^−^ level showed a greater decrease (− 0.6 vs. + 0.9 mEq/L, *P* < 0.01) in the saline group than in the balanced crystalloid group. Furthermore, a higher incidence of hyperchloremia was observed in the saline group than in the balanced crystalloid group (27 vs. 4%, *P* < 0.01). However, there were minimal changes in pH (− 0.01 vs. − 0.01, *P* = 0.99) and Cr-eGFR (− 1.5 vs. + 1.1 mL/min/1.73 m^2^, *P* = 0.96) in both groups (Table 3). In addition, when comparing pre- and post-biopsy values within each group, only serum chloride levels were significantly elevated after the biopsy in the saline group (T0 106.2 vs. T7 107.9 mEq/L, *P* < 0.01) (Supplementary Table 1). In the saline and balanced crystalloid groups, patients were categorized into those with an increase in AVP concentrations and those with no increase in AVP concentrations at T7 (Supplementary Table 2). There was no significant difference in the patients’ background information between those with and those without increased AVP concentrations within each group. In the saline group, an increase in AVP concentrations was observed in 20.6% (7/34) of patients, and in the balanced crystalloid group, it was observed in 24.0% (6/25) of patients. However, no cases of hyponatremia were observed, and there was no difference in serum sodium concentrations between those with and those without increased AVP concentrations (Supplementary Table 2). Finally, a comparison was conducted based on the presence or absence of PONV and pain (Table 4). PONV and pain were observed in 23.7% (14/59) of patients, and AVP concentrations in the PONV and pain positive group were significantly higher than those in the PONV and pain negative group at T7 (1.0 vs. 1.8 pg/mL, *P* = 0.04). However, there were no differences in serum sodium concentrations between the PONV and pain positive and PONV and pain negative groups at T7.

**Table 4:** Characteristics of patients with and without PONV and pain

	Negative (*n* = 45)	Positive (*n* = 14)	*P* value
Baseline			
Age (year)	13.0 (9.0 − 15.0)	10.0 (7.3 − 12.8)	0.18
Weight (kg)	42.1 (27.1 − 55.8)	33.2 (24.1 − 42.1)	0.17
Times of biopsy	2.3 (1 − 7)	2.0 (1 − 3)	0.52
T0 AVP (pg/mL)	0.8 (0.5 − 1.4)	1.35 (0.9 − 1.9)	0.09
T0 Na (mEq/L)	138.6 (135.0 − 142.0)	138.4 (136.0 − 140.0)	0.32
Blood test after kidney biopsy			
T7 AVP (pg/mL)	1.0 (0.5 − 1.5)	1.8 (1.2 − 5.2)	0.04
T7 AVP > 2.8 pg/mL (%)	18 (8/45)	43 (6/14)	0.06
T7 Na (mEq/L)	139.2 (137.0 − 142.0)	139.0 (137.0 − 140.0)	0.50
Change in Na (mEq/L)	0.4 (− 3.0 ~ 3.0)	0.6 (− 1.0 ~ 3.0)	0.50

*PONV*, postoperative nausea and vomiting; *AVP*, arginine vasopressin; *T0*, time zero; *T7*, time + 7 h

### Protocol deviations and adverse events

In this study, no significant adverse events were observed, and no patients required changes in the type or infusion rate of fluids. There were no patients of acute kidney injury following kidney biopsy, defined as a 1.5-fold increase in serum creatinine. Additionally, no patients experienced complications from the biopsy or fluid therapy resulting in an extended hospital stay.

## Discussion

This study investigated the changes in biochemical parameters between hydration therapy with saline and balanced crystalloids for pediatric patients who underwent kidney biopsies. We found several important findings. First, when the isotonic solutions saline and balanced crystalloids were used as hydration therapy, they prevented infusion-related hyponatremia. Second, we found that PONV and pain might be factors associated with the increase in AVP concentrations after a kidney biopsy. Additionally, the administration of saline resulted in increased serum chloride concentration, while pH and Cr-eGFR remained unchanged 5 h after the kidney biopsy.

This study showed that hydration therapy with saline and balanced crystalloids prevented hyponatremia in pediatric patients under hydration after a kidney biopsy. In recent years, the effectiveness of isotonic fluids has become widely recognized. Recent reviews of maintenance fluid therapy for children receiving inpatient care have reported that isotonic fluids are superior to hypotonic fluids in reducing the risk of hyponatremia [10]. However, few reports have focused on the effectiveness of isotonic fluids as hydration therapy for pediatric patients during moderately invasive procedures such as a kidney biopsy. In our previous study, a 21% incidence of hyponatremia was observed during hydration therapy in the hypotonic solutions group after kidney biopsies, whereas no cases of hyponatremia occurred in the saline group [6]. Similar results were obtained in this study in which the administration of saline and balanced crystalloids did not lead to the development of hyponatremia. In addition, we evaluated AVP concentrations. In our previous report, AVP concentrations were increased in 28% of patients after a kidney biopsy [6]. Among patients with elevated AVP concentrations, 60% of those who received hypotonic fluids developed hyponatremia, while no hyponatremia was observed in patients who received isotonic fluids [6]. In the current study, AVP concentrations were increased in 24% of patients, which is a similar rate to our previous study, but no cases of hyponatremia were observed. Therefore, this study indicated that the administration of isotonic fluids, namely saline and balanced crystalloids, could prevent the development of hyponatremia, despite the common occurrence of an elevation in AVP concentrations after a kidney biopsy.

PONV and pain could be factors that contributed to the elevation in AVP concentrations after a kidney biopsy. In our previous study, an elevation in AVP concentrations was observed in the PONV and pain positive group, while there were no cases of AVP elevation in the PONV and pain negative group [6]. In this study, AVP concentrations were significantly higher in the PONV and pain positive group than in the negative group at T7. However, even in the PONV and pain negative group, 15% (7/45) of patients had high AVP concentrations exceeding the normal range. These results indicate that PONV and pain may be associated with an elevation in AVP concentrations, and other factors may stimulate AVP secretion. Regarding the relationship between serum sodium concentrations and PONV and pain, a previous report showed that all patients who developed hyponatremia showed PONV and pain and elevated AVP concentrations [6]. However, in this study, no cases of hyponatremia were found even among PONV and pain positive cases. Furthermore, there was no difference in serum sodium concentrations or a change in serum sodium concentrations between PONV and pain positive and PONV and pain negative cases. Therefore, while PONV and pain may be a factor contributing to an elevation in AVP concentrations, PONV and pain did not affect serum sodium concentrations when administering isotonic fluids, including balanced crystalloids.

Saline infusion is known to be associated with hyperchloremia and acidosis, and hyperchloremia is also of concern due to its association with kidney function. A study comparing saline with balanced crystalloids in pediatric ICU patients found that chloride concentrations increased twice as much with saline infusion compared to balanced crystalloids [12]. Additionally, a randomized controlled trial in dehydrated adult patients, which compared saline with balanced crystalloids infusion, demonstrated that saline could exacerbate acidosis [17]. Furthermore, in a large, non-blinded cluster-randomized single-center trial comparing saline and balanced crystalloids in an adult ICU population, an association was reported between the administration of saline and an increased incidence of composite adverse kidney outcomes, including in-hospital mortality, new initiation of kidney replacement therapy (KRT), and acute kidney injury (AKI) [18]. This trend was also confirmed in non-critically ill patients in the same group [19]. However, the drawbacks of saline infusion are believed to depend on the situation. A double-blind, cluster-randomized, double-crossover trial comparing saline and balanced crystalloids in ICU patients found no significant differences in the incidence of AKI [20]. Furthermore, two recently reported large, multicenter, double-blind randomized trials comparing saline and balanced crystalloids in adult ICU patients showed no differences in the incidence of AKI or the need for KRT [21, 22]. These findings suggest that the effects of saline may vary depending on the situation, but many previous studies have focused on acutely or critically ill patients. In our study, which targeted relatively stable pediatric patients, the saline group showed a significant increase in chloride concentration and a significant decrease in HCO_3_^−^ compared with the balanced crystalloid group. However, there were no changes in pH or Cr-eGFR, indicating that saline infusion likely has no substantial clinical adverse effects.

### Strengths and limitations

To the best of our knowledge, there have been no reports comparing saline with balanced crystalloids as hydration therapy for pediatric patients who have a kidney biopsy. Although this study focused on kidney biopsies as the specific procedure, other interventions with similar levels of invasiveness and anesthesia use, such as bone marrow aspirates, lumbar punctures, and liver biopsies, may be associated with similar circumstances. In pediatric wards, such moderately invasive procedures for stable patients are common. Therefore, this study is important for evaluating the choice of fluid for pediatric patients, particularly the type of isotonic solution. Limitations of this study include the limited number of cases and the single-center design. Additionally, since the underlying causes for kidney biopsy varied among patients, the results may be influenced by these differences. Moreover, in our study, no cases of neonates or infants were included, so further evaluation is necessary in these patients with immature physiological function of the kidney. Furthermore, pain and nausea were assessed based on patient self-reporting without the use of objective measures such as pain scales, which may limit the accuracy of the evaluation.

## Conclusions

Saline and balanced crystalloids isotonic solutions used for hydration therapy in pediatric kidney biopsies have a low risk of hyponatremia. Although saline infusion increases serum chloride concentrations, it does not cause changes in pH or deterioration in kidney function, and these changes were clinically insignificant. Both saline and balanced crystalloids can be considered viable options for fluid therapy during pediatric kidney biopsy.

## Supplementary Information

Below is the link to the electronic supplementary material.

Graphical abstract (PPTX 78 KB)

Supplementary file2 (DOCX 70 KB)

## Data Availability

Data from this study can be obtained from the corresponding author upon reasonable request.
