# Supplementary material for: Saline versus balanced crystalloids for hydration post-kidney biopsy
Source: Pediatr Nephrol. 2024 Nov 25;40(4):1033–40. doi: 10.1007/s00467-024-06594-0 (PMC11885368; doi:10.1007/s00467-024-06594-0)
Supplement: Supplementary file 1 — Graphical abstract (PPTX 78 KB) [file 467_2024_6594_MOESM1_ESM.pptx]

## Slide 1
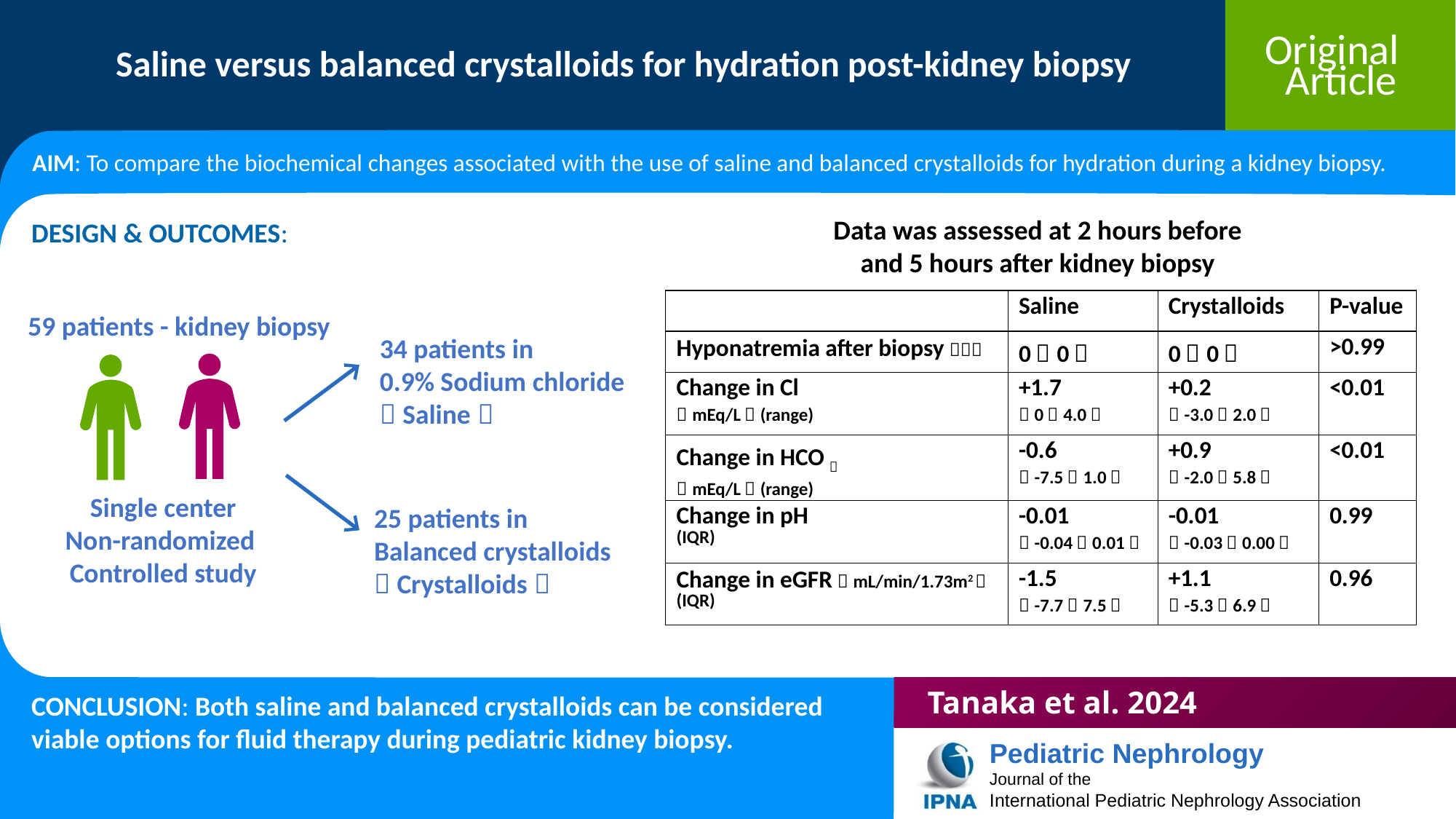

Saline versus balanced crystalloids for hydration post-kidney biopsy
AIM: To compare the biochemical changes associated with the use of saline and balanced crystalloids for hydration during a kidney biopsy.
Data was assessed at 2 hours before
and 5 hours after kidney biopsy
DESIGN & OUTCOMES:
| | Saline | Crystalloids | P-value |
| --- | --- | --- | --- |
| Hyponatremia after biopsy（％） | 0（0） | 0（0） | >0.99 |
| Change in Cl （mEq/L）(range) | +1.7 （0～4.0） | +0.2 （-3.0～2.0） | <0.01 |
| Change in HCO３ （mEq/L）(range) | -0.6 （-7.5～1.0） | +0.9 （-2.0～5.8） | <0.01 |
| Change in pH (IQR) | -0.01 （-0.04～0.01） | -0.01 （-0.03～0.00） | 0.99 |
| Change in eGFR（mL/min/1.73m2）(IQR) | -1.5 （-7.7～7.5） | +1.1 （-5.3～6.9） | 0.96 |
59 patients - kidney biopsy
34 patients in
0.9% Sodium chloride
（Saline）
Single center
Non-randomized
Controlled study
25 patients in
Balanced crystalloids
（Crystalloids）
Tanaka et al. 2024
CONCLUSION: Both saline and balanced crystalloids can be considered viable options for fluid therapy during pediatric kidney biopsy.
