# Supplementary material for: Saline versus balanced crystalloids for hydration post-kidney biopsy
Source: Pediatr Nephrol. 2024 Nov 25;40(4):1033–40. doi: 10.1007/s00467-024-06594-0 (PMC11885368; doi:10.1007/s00467-024-06594-0)
Supplement: Supplementary file 2 — Supplementary file2 (DOCX 70 KB) [file 467_2024_6594_MOESM2_ESM.docx]

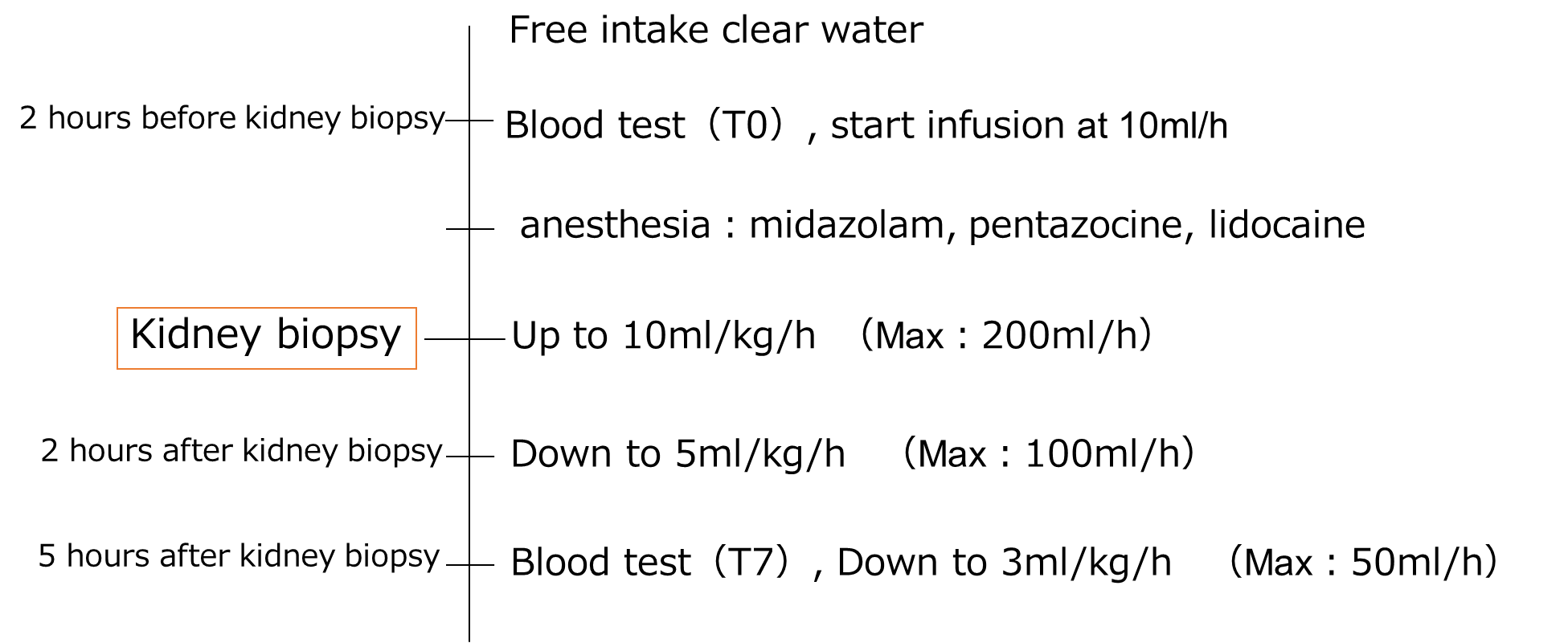
 **Supplementary Figure 1**

**Protocol of kidney biopsy and fluid therapy**. Fluid infusion was initiated at a rate of 10 mL/h starting 2 hours before the kidney biopsy. The rate was increased to 10 mL/kg/h immediately after the biopsy, adjusted to 5 mL/kg/h 2 hours post-biopsy, and then reduced to 3 mL/kg/h 5 hours post-biopsy. Blood tests were conducted 2 hours before and 5 hours after the biopsy.

**Supplementary Table 1**

| **Clinical parameters** | T0 | T7 | **P** |
| --- | --- | --- | --- |
| **0.9% sodium chloride group** |  |  |  |
| Na（mEq/L） | 138.7（135-140） | 139.4（137-141） | 0.052 |
| Cl（mEq/L） | 106.2(100-110) | 107.9（102-112） | <0.01 |
| HCO_3_^-^（mEq/L） | 23.4（16.9-26.7） | 22.9（16.7-27.3） | 0.36 |
| pH | 7.37（7.31-7.47） | 7.36（7.27-7.49） | 0.17 |
| Cr-eGFR（mL/min/1.73m^2^） | 104.3（95.0-118.1） | 99.3（82.9-119.7） | 0.63 |
| **Balanced crystalloids group** |  |  |  |
| Na（mEq/L） | 138.8(136-142) | 138.9(137-141) | 0.74 |
| Cl（mEq/L） | 106.0（102-109） | 106.2（102-110） | 0.66 |
| HCO_3_^-^（mEq/L） | 23.5（19.3-28.0） | 24.4（19.3-29.6） | 0.85 |
| pH | 7.38（7.32-7.50） | 7.36（7.33-7.46） | 0.15 |
| Cr-eGFR（mL/min/1.73m^2^） | 104.3（95.0-118.1） | 99.2（82.9-119.7） | 0.63 |

Cr Creatinine, eGFR estimated glomerular filtration rate, T0 time zero, T7 time +7 hours

| **Clinical parameters** | **Non-increased AVP** | **Increased AVP** | **P** |
| --- | --- | --- | --- |
| **0.9% sodium chloride group** | **AVP<2.8（n=26）** | **AVP>2.8（n=8）** | **P** |
| **Characteristic** |  |  |  |
| Age（year） | 10.5（7.0-14.8） | 13.0（10.8-15.0） | 0.24 |
| Weight（kg） | 29.7（22.1-48.3） | 50.7（43.6-56.4） | 0.07 |
| BUN/Cr T0 | 24.4（17.4-29.3） | 25.4（19.2-32.3） | 0.65 |
| Cr-eGFR（mL/min/1.73m^2^） | 104.0（84.0-121.7） | 105.4（99.2-110.1） | 1.0 |
| T0 Na（mEq/L）（range） | 138.8（135.0-141.0） | 136.5（137.0-140.0） | 0.70 |
| T0 AVP（pg/mL） | 0.7（0.5-1.0） | 1.4（1.3-2.1） | <0.01 |
| **Blood data after kidney biopsy** |  |  |  |
| T7 Na（mEq/L）（range） | 139.5（137.0-141.0） | 139.3（138.0-140.0） | 0.67 |
| T7 hyponatremia（<137mEq/L）［n（%）］ | 0（0） | 0（0） | 1.0 |
| Change in Na（mEq/L）（range） | 0.7（-2.0～3.0） | 0.8（-1.0～2.0） | 0.90 |
| T7 AVP（pg/mL） | 0.7（0.4-1.2） | 5.6（3.1-12.8） | <0.01 |
| **Balanced crystalloids group** | **AVP<2.8（n=19）** | **AVP>2.8（n=6）** | **P** |
| C**haracteristic** |  |  |  |
| Age（year） | 12.0（9.0-15.0） | 12.5（7.8-15.8） | 0.95 |
| Weight（kg） | 36.5（28.6-53.1） | 37.7（26.7-50.0） | 0.85 |
| BUN/Cr T0 | 23.6（15.7-30.6） | 23.7（16.1-27.4） | 0.98 |
| Cr-eGFR（mL/min/1.73m^2^） | 98.9（84.8-118.0） | 95.3（86.5-106.1） | 0.93 |
| T0 Na（mEq/L）（range） | 138.9（136.0-142.0） | 138.1（138.0-139.0） | 0.24 |
| T0 AVP（pg/mL） | 0.9（0.7-1.6） | 1.55（1.35-1.7） | 0.24 |
| **Blood data after kidney biopsy** |  |  |  |
| T7 Na（mEq/L）（range） | 139.1（137.0-141.0） | 138.3（137.0-139.0） | 0.11 |
| T7 hyponatremia（<137mEq/L）［n（%）］ | 0（0） | 0（0） | 1.0 |
| Change in Na（mEq/L）（range） | 0.16（-3.0～2.0） | 0.14（-1.0～1.0） | 0.97 |
| T7 AVP（pg/mL） | 1.0（0.6-1.2） | 15.5（8.3-27.5） | <0.01 |

**Supplementary Table 2**

Cr Creatinine, eGFR estimated glomerular filtration rate, AVP arginine vasopressin, T0 time zero, T7 time +7 hours
